# Refining the structure−activity relationships of 2-phenylcyclopropane carboxylic acids as inhibitors of O-acetylserine sulfhydrylase isoforms

**DOI:** 10.1080/14756366.2018.1518959

**Published:** 2018-10-26

**Authors:** Joana Magalhães, Nina Franko, Giannamaria Annunziato, Marco Pieroni, Roberto Benoni, Anna Nikitjuka, Andrea Mozzarelli, Stefano Bettati, Anna Karawajczyk, Aigars Jirgensons, Barbara Campanini, Gabriele Costantino

**Affiliations:** a P4T group, Department of Food and Drug, University of Parma, Parma, Italy;; b Laboratory of Biochemistry and Molecular Biology, Department of Food and Drug, University of Parma, Parma, Italy;; c Latvian Institute of Organic Synthesis, Riga, Latvia;; d National Institute of Biostructures and Biosystems, Rome, Italy;; e Institute of Biophysics, Pisa, Italy;; f Department of Neurosciences, University of Parma, Parma, Italy;; g Selvita S.A., Park Life Science, Kraków, Poland;; h Centro Interdipartimentale Misure (CIM)’G. Casnati’, University of Parma, Parma, Italy

**Keywords:** Antibacterials, cysteine, Gram-negatives, O-acetylserine sulfhydrylase, permeability

## Abstract

The lack of efficacy of current antibacterials to treat multidrug resistant bacteria poses a life-threatening alarm. In order to develop enhancers of the antibacterial activity, we carried out a medicinal chemistry campaign aiming to develop inhibitors of enzymes that synthesise cysteine and belong to the reductive sulphur assimilation pathway, absent in mammals. Previous studies have provided a novel series of inhibitors for O-acetylsulfhydrylase – a key enzyme involved in cysteine biosynthesis. Despite displaying nanomolar affinity, the most active representative of the series was not able to interfere with bacterial growth, likely due to poor permeability. Therefore, we rationally modified the structure of the hit compound with the aim of promoting their passage through the outer cell membrane porins. The new series was evaluated on the recombinant enzyme from *Salmonella enterica serovar* Typhimurium, with several compounds able to keep nanomolar binding affinity despite the extent of chemical manipulation.

## Introduction

The amino acid cysteine is the precursor of sulphur-containing biomolecules like Fe-S clusters, modified tRNAs, thiamine, biotin, and glutathione, which are essential components in bacteria for fitness and virulence. Moreover, in pathogens possessing long latent phases such as *Trichomonas vaginalis*, *Mycobacterium tuberculosis,* and *Salmonella enterica serovar* Typhimurium (hereafter *Salmonella*), sulphur-containing biomolecules are pivotal in surviving the hostile oxidative environment created by the host defences^1–4^. Interestingly, studies in macrophages as well as mouse models have demonstrated that the deletion of those genes involved in sulphur metabolism reduces both virulence and survival of *M. tuberculosis*, especially during chronic infection^1^ ^,^ ^2^ ^,^ ^4–6^. While mammals use sulphur-containing amino acids like methionine and cysteine from diet to fulfil sulphur supply, bacteria actively transport sulphate and thiosulphate and reduce sulphate to sulphide in a highly demanding energetic process. To this scope, O-acetylserine (OAS), synthesised by serine acetyl transferase (SAT), incorporates sulphide to produce cysteine in a β-replacement reaction catalysed by the pyridoxal 5'-phosphate (PLP)-dependent enzyme OAS sulfhydrylase (OASS). OASS is a well-studied enzyme that exists in two isoforms, namely OASS-A, -coded by *cysK,* and OASS-B, coded by *cysM*. The reaction mechanism of both isoforms is of a ping-pong type, in which OAS binds to the internal aldimine linkage between enzyme and PLP, forming an α-aminoacrylate intermediate with the release of acetate. In the second half reaction, bisulphide binds as the second substrate to the α-aminoacrylate and cysteine is released^1^ ^,^ ^7^ ^,^ ^8^.

OASS-A is a homodimer of 315 amino acid residues *per* subunit and the two active sites are located on opposite side, far from the dimer interface. Each subunit is made of two domains, a C-terminal and an N-terminal domain. In turn, each domain is composed of an α/β-fold with a central twisted β-sheet surrounded by α-helixes^8^. Concerning the folding, A and B isozymes follow a similar pattern, but in terms of active site the B isoform presents two additional acidic residues, which increase its overall charge^7^.

OASS-A is highly expressed at basal levels and together with SAT forms the cysteine synthase complex, whose function is still controversial^1–4^ ^,^ ^9^. Interestingly, SAT physiologically inhibits OASS activity through its carboxy-terminal portion by competing with the natural substrate OAS for binding to the active site.

**Figure 1.**
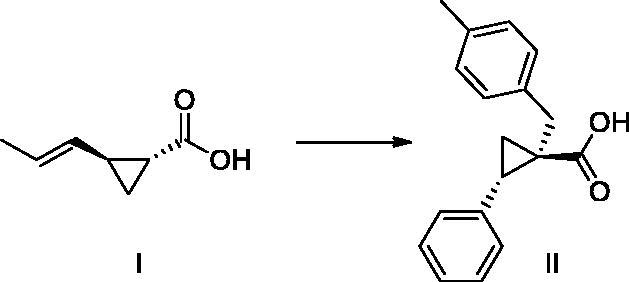
Chemical structure of compounds **I** and **II**.

Expression of genes coding for OASS-A and OASS-B have already been correlated with antibiotic resistance in *Salmonella*, being the knock-out strains more susceptible to antibiotics^2^ ^,^ ^4^ ^,^ ^10^ ^,^ ^11^. Given these findings, we have explored the potential of cysteine biosynthesis shutdown as a strategy to obtain new adjuvants of antibacterial agents^1^ ^,^ ^2^. The first peptide inhibitors of *Haemophilus influenzae* OASS (HiOASS) were designed based on the C-terminal sequence of SAT, which acts as the physiological inhibitor of OASS^12^. Later, peptidomimetic compounds were designed in which the carboxylic acid moiety and the hydrophobic motif of the final amino acid isoleucine were considered critical features for OASS binding, therefore both were kept as structural motives of small-molecule inhibitors (compound **I,** Figure 1). After these first speculative attempts, several rounds of optimisation of compound **I** led to the discovery of a number of derivatives, culminating with compound **II** (Figure 1), to our knowledge the most potent inhibitor of bacterial OASS described so far^13^.

Since *E.coli* CysK/CysM double mutant is a cysteine auxotroph^14^ ^,^ ^15^, compound **II** was evaluated in minimal medium against *E.coli*. Despite its high potency in the enzymatic assays, it failed to show appreciable inhibitory activity on cell growth. Further biological evaluation suggested poor permeability as the main reason for the lack of activity on bacterial cell growth, as the co-administration of sub-lethal concentrations of polymyxin B nonapeptide (PMBN), a permeabilizing agent, eventually led to a measurable MIC for this derivative [manuscript in preparation].

Poor permeability is considered most definitely one of the main hurdles in antibacterial drug discovery^16^ ^,^ ^17^. While hydrophobic drugs, such as macrolides, pass across the outer membrane lipid bilayer, small hydrophilic drugs, such as β-lactams, use preferentially porins to accumulate inside the cell^17–19^. Actually, difference in membrane composition, and therefore different permeability, is at the base of the antibacterial selectivity toward Gram-positive and Gram-negative bacteria. A retrospective analysis has shown that antibacterials, in general, present higher molecular weight and lower lipophilicity than non-antiinfective drugs. In addition, Gram-negative active compounds tend to present lower molecular weight and higher polarity compared to the agents active against Gram-positive bacteria^16^ ^,^ ^17^ ^,^ ^20^ ^,^ ^21^.

Several attempts to rationalise the properties that allow small molecule penetration in Gram-negative bacteria and therefore increase the success of antibacterial drug-discovery programmes have been made^21^ ^,^ ^22^. Quantification of drug accumulation with a set of compounds exhibiting different properties was performed, and these studies provided an observation that positively charged compounds possessing a non-sterically hindered amine, show higher permeability. Also rigidity, low globularity and amphiphilicity contributed significantly to enhance the compound penetration^16^. However, despite the numerous attempts made, there are not unequivocal rules available to predict the physicochemical properties required for the accumulation of a small molecule into the bacterial cell, therefore the chemical manipulation of hit compounds still remains the most valuable means to improve activity in cells. These considerations prompted us to carry out a ligand-based drug design approach for the synthesis of analogues of compound **II**.

## Methods

### Synthetic chemistry

Compounds **6**, **7,** and **14** were synthesised using an already reported protocol^13^. The reaction of phosphonoacetate **2** with styrene oxides allowed the stereoselective synthesis of the required stereodefined *trans*-R-phenyl-1-(4-methylbenzyl)cyclopropane-1-carboxylate esters **6**, **7**, and **14**. On a similar vein, Wittig reaction between phosphonoacetate **2** and the proper aldehydes provided intermediates **3**, **31**, and **39–42**, that were cyclopropanated under Corey–Chaykovsky conditions to give the corresponding esters **4**, **32** and **43**–**46**. Styrene oxides, when not commercially available, were synthesised from the styrene or the aldehyde derivatives in good overall yields. Nucleophilic substitution of chloride with *i*-propylamine in intermediate **7** gave an ester **9** which was hydrolysed to acid **10**, whereas catalytic hydrogenation of nitro group in intermediate **6** and subsequent hydrolysis of ester **11** provided acid **12**.

Hydrolysis of ester **14** gave raise to key intermediate acid **15**, that was used as key intermediate for the synthesis of amides **16**–**22** using standard amide bond forming conditions. Carboxylic acid **15** was also subjected to Curtius rearrangement in the presence of diphenylphosphoryl azide, triethylamine, and using *tert-*butanol as a solvent. The resulting Boc-amine **23** was deprotected under acidic conditions and transformed to sulphonamides **24** and **25**.

Tetrazole analogue **29** was prepared in two steps from diethyl (1-cyano-2-(p-tolyl)ethyl) phosphonate **27**. The reaction of phosphonoacetate **27** with styrene oxide provided nitrile **28** which was subjected to the tetrazole forming reaction with sodium azide.

Pyridylmethyl substituted carboxylic acid **33** was prepared starting form triethyl phosphonoacetate (**1**). This was transformed to ethyl 2-(diethoxyphosphoryl)-3-(pyridin-4-yl) propanoate (**30**) by alkylation with 4-(bromomethyl) pyridine in the presence of sodium hydride. The reaction of intermediate **30** with benzaldehyde provided substrate **31** for Corey–Chaykovsky cyclopropanation that gave ester **32**. The condensation of triethyl phosphonoacetate (**1**) with paraformaldehyde gave unsaturated phosphonoacetate **34** which was subjected to Michael addition with a set of heterocycles, affording compounds **35**–**38**. Horner–Wadsworth–Emmons reaction of these intermediates gave the corresponding acrylates **39–42**, that were subjected to cyclopropanation using Corey–Chaykovsky conditions, as described above.

All of the title carboxylic acids herein reported, were obtained after hydrolysis of the corresponding ethyl ester with lithium hydroxide.

### Protein preparation

StOASS-A and StOASS-B, expressed in *E. coli,* were purified by metal affinity chromatography as described previously^13^. Proteins were more that 95% pure based on SDS-PAGE analysis (data not shown).

### Determination of dissociation constants

Ligand affinity was measured by direct fluorimetric titrations, monitoring the fluorescence emission spectrum of cofactor PLP upon excitation at 412 nm. 0.1–1 µM OASS was titrated by increasing concentrations of ligand in 100 mM HEPES pH 7.0, 1% DMSO, at 20 °C. The dependence of fluorescence emission intensity on the concentration of compound was fitted to a binding isotherm:

$$I=I_{0}+\frac{\Delta I\cdot [\text{ligand}]}{K_{d}+[\text{ligand}]}$$

where, *I* is the fluorescence emission intensity at a given ligand concentration, *I*_0_ is the fluorescence emission intensity in the absence of ligand, Δ*I* is the amplitude and *K*_d_ is the dissociation constant. When the *K*_d_ was lower than the concentration of the protein used in the assay, the dependence of the fluoresecnce emission intensity on ligand concentration was fitted to a quadratic equation that describes tight binding^23^. The fluorescence competitive binding assay, described elsewhere^24^, was used to evaluate whether compounds bind at the active site of the enzyme or to an allosteric site. The assay is based on the formation of a low-affinity, highly fluorescent complex between OASS and 1-ethylcyclopropane-1,2-dicarboxylic acid. Binding of a higher affinity compound to the active site displaces 1-ethylcyclopropane-1,2-dicarboxylic acid and leads to a decrease in the fluorescence emission intensity.

### Activity assays

Enzyme activity under steady-state conditions was measured by a discontinuous method as described previously^23^. Briefly, the reaction was carried out in 100 mM HEPES pH 7.0 in the presence of 6 nM OASS, 60 nM BSA to prevent enzyme adhesion to tube wall, 1 mM OAS (equal to the *K*_m_ value of this subsrate), 0.6 mM Na_2_S (a saturating concentration), 1% DMSO and variable concentrations of inhibitor. Reaction was stopped at time intervals by the addition of acetic acid and concentration of cysteine was determined by a modification of the ninhydrin assay^25^. The fractional velocity as a function of inhibitor concentration was determined and IC_50_ was calculated by the following equation:

$$\frac{v_{i}}{v_{0}}=\frac{1}{1+(\frac{\left[I\right]}{{IC}_{50}})}$$

where, *v*_0_ is the reaction velocity in the absence of inhibitor and *v*_i_ is reaction velocity in the presence of inhibitor at concentration [I].

For a competitive inhibitor against OAS the IC_50_ measured under these conditions is about 2⋅*K*_d_ ^26^.

### MIC determination

Minimal inhibitory concentration assays were performed according to the CLSI guidelines (Clinical and Laboratory Standards Institute; Performance Standards for Antimicrobial Susceptibility Testing; Twenty-Fourth Informational Supplement; CLSI document M100-S24, CLSI; 2014.). The compounds were dissolved in DMSO to obtain a stock solution 100-fold more concentrated than the desired higher concentration and then 10 serial dilutions were performed. One microliter of each dilution was transferred to the microtiter plate.

*Escherichia coli* ATCC25922 was grown until log phase on liquid Mueller–Hinton broth at 37 °C in a shaker at 225 rpm. Bacterial suspension was then centrifuged at 2500 rpm for 15 min and diluted with phosphate buffer to obtain a solution with a turbidity of about 0.5 McFarland. Bacterial suspension was further diluted (1:100) with fresh LB 20%. Fifty microliters of the bacterial suspension containing 10^6^ CFU/mL were inoculated into each well, to obtain a final concentration of 5 × 10^5^ CFU/mL. Each plate contained growth control wells and sterility check wells. The plates were incubated at 37 °C without agitation during 16h–24h and MIC was determined.

## Experimental

### General information

All the reagents were purchased from Sigma-Aldrich (St. Louis, MO), Alfa-Aesar (Haverhill, MA), or Fluorochem (Hadfield, UK) at reagent purity and, unless otherwise noted, were used without any further purification. Dry solvents used in the reactions were obtained by distillation of technical grade materials over appropriate dehydrating agents. MCRs were performed using CEM Microwave Synthesizer-Discover model. Reactions were monitored by thin layer chromatography on silica gel-coated aluminium foils (silica gel on Al foils, SUPELCO Analytical, Sigma-Aldrich) at 254 and 365 nm. Where indicated, intermediates and final products were purified by silica gel flash chromatography (silica gel, 0.040−0.063 mm), using appropriate solvent mixtures. ^1^H NMR and ^13^ C NMR spectra were recorded on a BRUKER AVANCE spectrometer at 400 and 100 MHz, respectively, with TMS as internal standard. 1H NMR spectra are reported in this order: multiplicity and number of protons. Standard abbreviation indicating the multiplicity were used as follows: s: singlet, d: doublet, dd: doublet of doublets, t: triplet, q: quadruplet, m: multiplet, and br: broad signal. HPLC/MS experiments were performed with an Agilent 1100 series HPLC apparatus, equipped with a Waters Symmetry C18, 3.5 μm, 4.6 mm × 75 mm column and an MS: Applied Biosystem/MDS SCIEX instrument, with API 150EX ion surce. Analytical, preparative HPLC and Electron Spray Ionization condition (ESI) mass spectra were performed on an Agilent uHPLC (1290 Infinity) and an Agilent Prep-HPLC (1260 Infinity) both equipped with a Diode Array Detector and a Quadrupole MS Dusing mixture gradients of Formic acid/water/acetonitrile as system solvent. HRMS experiments were performed with an LTQ ORBITRAP XL THERMO apparatus. All compounds were tested as 95% purity or higher (by HPLC/MS).

**Ethyl 2-(diethoxyphosphoryl)-3-(*p*-tolyl)propanoate (2)**. Ethyl 2-(diethoxyphosphoryl)acetate (1 g, 4.5 mmol) was added dropwise to a cooled suspension of NaH (196 mg, 4.9 mmol) in dry DME (9 ml). After stirring at room temperature for 2 h, 1-(bromomethyl)-4-methylbenzene (910 mg, 4.9 mmol) was added, and the mixture was stirred at 60 °C for additional 2 h. After quenching with water, the mixture was extracted with ethyl acetate (3 × 50 ml), and the combined organic layers were washed with brine, dried over Na_2_SO_4_ and concentrated under reduced pressure. The crude material was purified through flash chromatography using ethyl acetate/petroleum ether (3:7) to give the title compound as colourless oil in 75% yield. ^1^H-NMR (300 Mhz-CDCl_3_): δ = 7.2 − 7.0 (m, 4 H), 4.2 − 4.1 (m, 6H), 3.3 − 3.1 (m, 3 H), 2.3 (s, 3 H), 1.4 − 1.1 (m,9H).

Following a similar procedure, but using (Bromomethyl)pyridine hydrobromide in place of 1-(bromomethyl)-4-methylbenzene, and THF/DMF (1:1) as the solvent, compound **30** was obtained.

**Ethyl 2-(diethoxyphosphoryl)-3-(pyridin-4-yl)propanoate (30).** Purification with acetone/ethyl acetate (15:85 → 20:80) afforded the desired compound as a yellow oil in 20% yield. ^1^H NMR (300 MHz, CDCl_3_) δ 8.50 (d, *J* = 5.7 Hz, 2H), 7.14 (d, *J* = 6.0 Hz, 2H), 4.36 − 3.94 (m, 6H), 3.42 − 3.06 (m, 3H), 1.35 (t, *J* = 7.1 Hz, 6H), 1.16 (t, *J* = 7.1 Hz, 3H).

**General procedure for the Wittig-Horner reaction (synthesis compounds 6, 7, and 14).** *n*-BuLi (2 equiv) was added dropwise to a solution of compound **2** (2 equiv) in dry DME (2.5 ml/mmol), stirring under nitrogen at room temperature. After 30 min, the proper styrene oxide (1 equiv) was added in one portion. The reaction was stirred at 90 °C for about 18 h and then quenched with the addition of saturated NH_4_Cl aqueous Solution (8 ml). The product was extracted with ethyl acetate (3 × 20 ml) and the combined organic layers were separated, washed with brine, dried over anhydrous Na_2_SO_4_, and concentrated under reduced pressure to yield a residue that is purified by flash chromatography on silica gel. Yields, purification methods and other analytical data are reported below.

**Ethyl 1-(4-methylbenzyl)-2-(4-nitrophenyl)cyclopropane-1-carboxylate (6).** The product was obtained as a yellow oil in 9% yield after purification by flash column chromatography using ethyl acetate/petroleum ether (1:99) as eluent. ^1^H NMR (300 MHz, CDCl_3_) δ 8.18 (d, *J* = 8.8 Hz, 2H), 7.39 (d, *J* = 8.4 Hz, 2H), 7.00 (q, *J* = 8.1 Hz, 4H), 4.15 (tt, *J* = 7.1, 3.5 Hz, 2H), 3.12 (d, *J* = 15.6 Hz, 1H), 2.99 − 2.82 (m, 1H), 2.29 (s, 3H), 2.08 − 1.88 (m, 2H), 1.47 (dd, *J* = 7.2, 5.3 Hz, 1H), 1.21 (t, *J* = 7.1 Hz, 3H).

**Ethyl 2-(4-(chloromethyl)phenyl)-1-(4-methylbenzyl)cyclopropane-1-carboxylate (7).** Purification by flash column chromatography on silica gel using ethyl acetate/petroleum ether (1:9 9 → 3:97) afforded the product as a colourless oil in 30% yield. ^1^H NMR (400 MHz, CDCl_3_) δ 7.39 (d, *J* = 7.5 Hz, 2H), 7.27 (d, *J* = 7.9 Hz, 2H), 7.06 (s, 4H), 4.62 (s, 2H), 4.25 − 4.09 (m, 2H), 3.20 (d, *J* = 15.4 Hz, 1H), 2.86 (t, *J* = 7.9 Hz, 1H), 2.32 (s, 3H), 1.98 (d, *J* = 15.6 Hz, 2H), 1.49 − 1.38 (m, 1H), 1.24 (t, *J* = 7.1 Hz, 3H).

**(1R,2S)-1-Ethyl 1-(4-methylbenzyl)-2-phenylcyclopropane-1-carboxylate (14).** Flash chromatography on silica gel using ethyl acetate/petroleum ether (1:99) afforded the product as yellow oil in 65% yield. ^1^H NMR (300 MHz, CDCl_3_) δ 7.30 (dddd, *J* = 13.4, 11.6, 7.5, 3.7 Hz, 5H), 7.03 (s, *J* = 7.9 Hz, 4H), 4.21 − 4.08 (m, 2H), 3.22 − 3.06 (m, 1H), 2.86 − 2.77 (m, 1H), 2.29 (s, 3H), 1.91 − 1.83 (m, 2H), 1.40 (dd, *J* = 7.2, 5.0 Hz, 1H), 1.21 (t, *J* = 7.1 Hz, 3H).

**Ethyl 2-(4-((isopropylamino)methyl)phenyl)-1-(4-methylbenzyl)cyclopropane-1-carboxylate (9).** Isopropylamine (26 µL, 0.292 mmol) was added dropwise to compound **7** (50 mg, 0.15 mmol) in neat at 0 °C, and the mixture was allowed to stir at room temperature for 24 h. The reaction was quenched with the addition of 4 M NaOH aq. solution, extracted with dichloromethane (3 × 5 ml), and the combined organic layers were separated, washed with brine, dried over anhydrous Na_2_SO_4_, and concentrated under reduced pressure. The residue obtained was purified by flash chromatography on silica gel using methanol/dichloromethane (2:98) to obtain the title compound as a colourless oil in 60% yield. ^1^H NMR (300 MHz, CDCl_3_) δ 7.31 (t, *J* = 7.9 Hz, 2H), 7.22 (d, *J* = 8.0 Hz, 2H), 7.05 (s, 4H), 4.27 − 4.06 (m, 2H), 3.81 (s, 2H), 3.19 (d, *J* = 15.6 Hz, 1H), 2.87 (ddd, *J* = 16.3, 13.4, 7.1 Hz, 2H), 2.31 (s, 3H), 1.93 − 1.79 (m, 2H), 1.40 (dd, *J* = 7.2, 5.0 Hz, 1H), 1.22 (t, *J* = 7.1 Hz, 3H), 1.14 (d, *J* = 6.3 Hz, 6H).

**Ethyl 2-(4-aminophenyl)-1-(4-methylbenzyl)cyclopropane-1-carboxylate (11).** Pd/C (18 mg) and triethylsilane (73 µL, 0.46 mmol) were added portion-wise to a solution of compound **6** (44 mg, 0.13 mmol) in dry methanol (6 ml), and the reaction mixture was stirred at room temperature under argon until complete consumption of the starting material. After filtration through a plug of celite, the organic layers were washed with brine, dried over anhydrous sodium sulphate, and concentrated under reduced pressure to afford the target compound as a yellow oil in quantitative yield. ^1^H NMR (400 MHz, MeOD) δ 7.05 − 6.97 (m, 6H), 6.72 (d, *J* = 8.5 Hz, 2H), 4.75 (s, 2H), 4.10 (dq, *J* = 11.0, 3.5 Hz, 2H), 3.06 (d, *J* = 15.5 Hz, 1H), 2.68 (dd, *J* = 9.2, 7.3 Hz, 1H), 2.27 (s, 3H), 1.98 (d, *J* = 15.5 Hz, 1H), 1.74 (ddd, *J* = 9.2, 4.9, 1.3 Hz, 1H), 1.38 (dd, *J* = 7.2, 4.9 Hz, 1H), 1.19 (t, *J* = 7.1 Hz, 3H).

**General procedure for the synthesis amides 16**–**22.** To a solution of 1-(4-methylbenzyl)-2-phenylcyclopropanecarboxylic acid (1 equiv) in dry DMF (20 ml/mmol), *N*-ethyldiisopropylamine (5 equiv), 1-hydroxybenzotriazole hydrate (1.5 equiv) and N-(3-Dimethylaminopropyl)-N′-ethylcarbodiimide hydrochloride (2 equiv) were added in one portion. The reaction mixture was stirred at room temperature for 30 min and then the proper amine (2 equiv) was added. Afterwards, reaction mixture was heated at 50 °C overnight. Volatiles were evaporated and the residue was solubilised in acetonitrile (1 ml), filtered and purified by HPLC. Yields and other analytical data are reported below.

***(1R,2S)*-1-N-methyl-1-(4-methylbenzyl)-2-phenyl-N-(tetrahydro-2H-pyran-4-yl)cyclopropane-1-carboxamide (16).** Colourless oil in 45% yield.^1^H NMR (400 MHz, CDCl_3_) δ 7.41 − 7.32 (m, 2H), 7.31 − 7.25 (m, 3H), 7.02 − 6.94 (m, 2H), 6.87 (s, 2H), 4.50 (s, 1H), 3.99 (d, *J* = 8.8 Hz, 2H), 3.44 (t, *J* = 12.3 Hz, 2H), 2.77 (s, 4H), 2.51 − 2.43 (m, 1H), 2.38 (d, *J* = 14.3 Hz, 1H), 2.26 (s, 3H), 1.77 − 1.58 (m, 3H), 1.36 (dd, *J* = 6.6, 5.9 Hz, 1H), 0.83 (dd, *J* = 6.5, 3.3 Hz, 2H).^13^C NMR (101 MHz, CDCl_3_) δ 179.7, 147.7, 136.6, 135.7, 131.7, 128.6, 128.5, 128.2, 126.4, 67.1, 59.8, 36.9, 29.5, 27.8, 25.4, 22.5, 20.8, 13.9. HRMS (ESI): calculated for C_24_H_30_O_2_N [M + H] 364.22711 found 364.22717.

***(1R,2S)*-1-N-((1-ethylpyrrolidin-2-yl)methyl)-1-(4-methylbenzyl)-2-phenylcyclopropane-1-carboxamide (17).** White oil in 34% yield. ^1^H NMR (400 MHz, CDCl_3_) δ 8.55 (s, 1H), 7.35 − 7.20 (m, 4H), 7.16 − 6.95 (m, 5H), 3.54 − 3.35 (m, 3H), 3.21 − 3.07 (m, 1H), 2.70 − 2.52 (m, 3H), 2.31 − 2.17 (m, 4H), 2.10 − 1.44 (m, 6H), 1.44 − 1.30 (m, 1H), 1.23 − 1.11 (m, 2H), 1.07 − 0.91 (m, 2H). ^13 ^C NMR (101 MHz, CDCl_3_) δ 168.8, 136.9, 136.1, 135.1, 129.0, 128.7, 128.6, 128.1, 126.5, 53.2, 52.8, 40.7, 33.8, 33.2, 31.1, 30.4, 28.1, 23.0, 20.7, 17.1, 10.4. HRMS (ESI): calculated for C_25_H_32_ON_2_ [M + H] 377.25874 found 377.25821.

***(1R,2S)*-1-N-benzyl-1-(4-methylbenzyl)-2-phenylcyclopropane-1-carboxamide (18).** Pearl powder in 55% yield. ^1^H NMR (400 MHz, CDCl_3_) δ 7.42 − 7.17 (m, 8H), 7.05 (dd, *J* = 18.9, 8.1 Hz, 4H), 6.92 − 6.77 (m, 2H), 5.84 (s, 1H), 4.43 (dd, *J* = 15.0, 5.8 Hz, 1H), 4.28 (dd, *J* = 15.0, 4.9 Hz, 1H), 3.04 − 2.90 (m, 1H), 2.83 (d, *J* = 17.5 Hz, 1H), 2.38 − 2.20 (m, 4H), 1.99 (dd, *J* = 8.6, 4.1 Hz, 1H), 1.44 (dd, *J* = 7.0, 4.7 Hz, 1H). ^13 ^C NMR (101 MHz, CDCl_3_) δ 173.8, 138.4, 137.7, 136.6, 135.9, 129.8, 129.6, 128.8, 128.7, 128.3, 127.6, 127.5, 127.1, 44.3, 34.6, 31.0, 30.8, 21.4, 18.9. HRMS (ESI): calculated for C_25_H_28_ON [M + H] 356.20089 found 356.20071.

***(1R,2S)*-1-(4-methylbenzyl)-2-phenyl-N-(thiophen-2-ylmethyl)cyclopropane-1-carboxamide (19).** Pearl powder in 56% yield. ^1^H NMR (400 MHz, CDCl_3_) δ 7.37  −  7.21 (m, 5H), 7.15 (d, *J* = 5.1 Hz, 1H), 7.03 (q, *J* = 8.2 Hz, 4H), 6.92 − 6.81 (m, 1H), 6.65 (d, *J* = 3.1 Hz, 1H), 5.89 (s, 1H), 4.52 (d, *J* = 4.4 Hz, 2H), 2.94 (t, *J* = 8.1 Hz, 1H), 2.83 (d, *J* = 17.3 Hz, 1H), 2.32 − 2.23 (m, 4H), 1.96 (dd, *J* = 9.1, 4.7 Hz, 1H), 1.47 − 1.36 (m, 1H). ^13 ^C NMR (101 MHz, CDCl_3_) δ 173.8, 141.4, 137.7, 136.6, 135.7, 129.8, 129.6, 128.7, 128.4, 127.2, 127.1, 125.8, 125.2, 39.2, 34.5, 31.3, 30.8, 21.4, 18.8. HRMS (ESI): calculated for C_23_H_24_ONS [M + H] 362.15731 found 362.15735.

***(1R,2S)*-1-N-((1-methyl-1H-imidazol-2-yl)methyl)-1-(4-methylbenzyl)-2-phenylcyclopropane-1-carboxamide (20).** Yellow oil in 52% yield.^1^H NMR (400 MHz, CDCl_3_) δ 8.33 (s, 1H), 7.28 (tdd, *J* = 7.7, 4.9, 3.7 Hz, 5H), 7.03 − 6.88 (m, 5H), 6.77 (d, *J* = 1.4 Hz, 1H), 4.47 (dd, *J* = 5.7, 1.9 Hz, 2H), 3.55 (d, *J* = 16.5 Hz, 3H), 2.96 (d, *J* = 16.8 Hz, 1H), 2.79 − 2.67 (m, 1H), 2.26 (s, *J* = 8.6 Hz, 3H), 2.18 (d, *J* = 16.8 Hz, 1H), 1.94 (ddd, *J* = 9.1, 5.0, 1.3 Hz, 1H), 1.38 (dd, *J* = 7.0, 5.0 Hz, 1H). ^13 ^C NMR (101 MHz, CDCl_3_) δ 173.8, 167.0, 144.9, 137.1, 135.8, 129.3, 129.1, 128.4, 128.1, 126.8, 125.3, 121.3, 34.7, 34.1, 33.2, 31.2, 30.8, 21.0, 17.4. HRMS (ESI): calculated for C_23_H_26_N_3_O [M + H] 360.20704 found 360.20645.

***(1R,2S)*-1-(4-methylbenzyl)-2-phenyl-N-(prop-2-yn-1-yl)cyclopropane-1-carboxamide (21).** Pearl powder in 57% yield. ^1^H NMR (400 MHz, CDCl_3_) δ 7.34 − 7.28 (m, 2H), 7.26 − 7.20 (m, 2H), 7.13 − 6.97 (m, 5H), 5.73 (s, 1H), 3.93 (tddd, *J* = 32.3, 27.2, 5.3, 2.5 Hz, 2H), 3.02 − 2.78 (m, 2H), 2.30 (d, *J* = 3.3 Hz, 4H), 2.13 (t, *J* = 2.5 Hz, 1H), 1.93 (ddd, *J* = 9.1, 4.7, 1.3 Hz, 1H), 1.41 (dd, *J* = 7.1, 4.8 Hz, 1H). ^13^ C NMR (101 MHz, CDCl_3_) *δ* 173.6, 137.2, 136.4, 135.3, 129.6, 129.3, 128.9, 128.4, 128.1, 126.9, 79.6, 71.4, 33.9, 31.1, 29.8, 21.1, 18.6. HRMS (ESI): calculated for C_21_H_22_ON [M + H] 304.16959 found 304.16946.

***(1R,2S)*-1-(4-methylbenzyl)-2-phenyl-N-((tetrahydrofuran-2-yl)methyl)cyclopropane-1-carboxamide (22).** White oil in 51% yield. ^1^H NMR (400 MHz, CDCl_3_) δ 7.40 − 7.17 (m, 5H), 7.16 − 6.96 (m, 4H), 5.93 (s, 1H), 3.69 − 3.50 (m, 2H), 3.45 − 3.30 (m, 2H), 3.21 (d, *J* = 13.6 Hz, 1H), 2.93 − 2.79 (m, 2H), 2.36 − 2.19 (m, 4H), 2.01 (dd, *J* = 8.2, 4.7 Hz, 1H), 1.87 (dd, *J* = 9.0, 4.6 Hz, 1H), 1.80 − 1.65 (m, 2H), 1.39 (ddd, *J* = 10.4, 7.6, 4.0 Hz, 2H). ^13^ C NMR (101 MHz, CDCl_3_) δ 173.4, 128.9, 128.9, 128.8, 127.9, 127.8, 127.6, 126.4, 67.7, 42.8, 42.1, 33.8, 30.3, 27.9, 27.4, 25.4, 25.3, 20.7, 18.0. HRMS (ESI): calculated for C_23_H_28_O_2_N [M + H] 350.21146 found 350.21121.

***(1R,2S)*-1-Tert-butyl (1-(4-methylbenzyl)-2-phenylcyclopropyl)carbamate (23).** Dry triethylamine (300 µL, 2.2 mmol) and diphenylphosphoryl azide (365 µL, 1.7 mmol) were added to a solution of compound **15** (409 mg, 1.5 mmol) in dry *t*-BuOH (5 ml). After stirring overnight at 90 °C under nitrogen atmosphere, the reaction mixture was then concentrated under vacuum and gently poured into a 10% Na_2_CO_3_ aq. solution. The mixture was extracted with Et_2_O (3 × 40 ml) and the organic layers separated, washed with brine, dried over anhydrous Na_2_SO_4_ and concentrated under reduced pressure, to give a black pitch that was purified by flash chromatography on silica gel using ethyl acetate/petroleum ether (1:9 9 → 3:97). The title compound was obtained as a yellowish oil in 50% yield. ^1^H NMR (400 MHz, CDCl_3_) δ 7.35 (t, *J* = 36.4 Hz, 5H), 7.06 (d, *J* = 6.6 Hz, 2H), 6.91 (d, *J* = 6.9 Hz, 2H), 4.82 (s, 1H), 2.97 (d, *J* = 13.4 Hz, 1H), 2.60 − 2.43 (m, 1H), 2.32 (s, 3H), 2.12 − 1.93 (m, 2H), 1.49 (s, *J* = 53.5 Hz, 9H), 1.14 (d, *J* = 6.0 Hz, 1H).

**General procedure for the preparation of sulphonamides 24 and 25.** Trifluoroacetic acid (55 equiv) was added dropwise to a solution of tert-butyl (1-(4-methylbenzyl)-2-phenylcyclopropyl)carbamate (1 equiv) in dichloromethane (18 ml/mmol) kept at 0 °C, and the reaction mixture was stirred at the same temperature for 1 h. After removal of the solvent in vacuum, the residue was dissolved in dry dichloromethane (7 ml/mmol) and cooled to −15 °C under nitrogen atmosphere. Dry triethylamine (3 equiv) and the proper sulphonyl chloride (1 equiv) were then added, and, after stirring at −15 °C for 1 h, additional triethylamine (3 equiv) and sulphonyl chloride (0.1 equiv) were added dropwise. After 90 min, the reaction was quenched with the addition of water (2 ml), and the organic layers were separated, washed with brine, dried over anhydrous Na_2_SO_4_, filtrated and concentrated under reduced pressure to obtain a residue that is purified by flash column chromatography on silica gel using ethyl acetate/petroleum ether (9:91). Yields, purification methods and other analytical data are reported below.

**(1R,2S)-1-N-(1-(4-methylbenzyl)-2-phenylcyclopropyl)methanesulfonamide (24).** The product was obtained as a pearl powder in 61% yield. ^1^H NMR (400 MHz, CDCl_3_) δ 7.49 − 7.32 (m, 4H), 7.31 (dd, *J* = 18.9, 11.8 Hz, 1H), 7.09 (dd, *J* = 21.7, 7.7 Hz, 4H), 4.76 (s, 1H), 3.02 (d, *J* = 15.2 Hz, 4H), 2.85 (t, *J* = 8.3 Hz, 1H), 2.33 (s, 3H), 2.13 (d, *J* = 14.8 Hz, 1H), 1.43 − 1.33 (m, 2H). ^13 ^C NMR (101 MHz, CDCl_3_) δ 136.7, 136.3, 134.5, 129.4, 129.3, 129.2, 128.4, 126.9, 44.0, 41.9, 38.9, 29.8, 21.0, 18.1. HRMS (ESI): calculated for C_18_H_21_O_2_NS [M + H] 316.13658 found 316.13721.

**(1R,2S)-4-methyl-N-(1-(4-methylbenzyl)-2-phenylcyclopropyl)benzenesulfonamide (25).** The product was obtained as a white powder in 50% yield. ^1^H NMR (400 MHz, CDCl_3_) δ 7.81 (d, *J* = 8.3 Hz, 2H), 7.43 − 7.23 (m, 7H), 7.06 (d, *J* = 7.9 Hz, 2H), 6.87 (d, *J* = 7.9 Hz, 2H), 4.94 (s, 1H), 2.88 (d, *J* = 14.6 Hz, 1H), 2.64 (dt, *J* = 15.5, 7.8 Hz, 1H), 2.45 (s, 3H), 2.33 (s, 3H), 1.93 (s, 1H), 1.35 − 1.19 (m, 2H). ^13 ^C NMR (101 MHz, CDCl_3_) δ 143.4, 139.6, 137.0, 136.1, 134.5, 129.7, 129.3, 129.2, 129.1, 128.3, 127.1, 126.7, 41.8, 38.2, 28.8, 21.5, 21.0, 17.8. HRMS (ESI): calculated for C_25_H_25_O_2_NS [M + H] 392.16788 found 414.15045 [M + Na].

**Ethyl 2-(diethoxyphosphoryl)acrylate (34).** Triethyl phosphonoacetate (2.23 mmol, 440 µL) was added to a suspension of paraformaldehyde (4.46 mmol, 138 mg) in piperidine (0.02 mmol, 22 µL) and methanol (5.4 ml) at 80 °C, and the reaction mixture was stirred at the same temperature for about 36 h until consumption of the starting material. The solvent was then evaporated under reduced pressure, and the residue obtained was taken up in toluene (1 ml/mmol), treated with *p*-toluenesulfonic acid monohydrate (0.22 mmol, 38 mg), and refluxed in a Dean-stark apparatus for 16 h. After removal of the solvent, the desired compound was obtained as a yellow-brownish oil in quantitative yield and used in the next reaction step without further purification. ^1^H NMR (400 MHz, CDCl_3_) δ 6.98 (dd, *J* = 42.0, 1.7 Hz, 1H), 6.74 (dd, *J* = 20.4, 1.7 Hz, 1H), 4.41 − 4.03 (m, 6H), 1.32 (tdd, *J* = 13.6, 7.9, 5.6 Hz, 9H).

**General procedure for the synthesis of phosphonoacetates 35**–**38.** The desired heterocycle (1.1 equiv) was added to a solution of ethyl 2-(diethoxyphosphoryl)acrylate (1 equiv) in triethylamine (1 equiv). The solution gradually turned pale yellow and after 15 min at room temperature the starting material was completely consumed and the volatiles were evaporated to dryness. The crude material obtained was purified by flash column chromatography on silica gel using a gradient of ethyl acetate in petroleum ether to afford the desired compounds. Yields, purification methods and other analytical data are reported below.

**Ethyl 2-(diethoxyphosphoryl)-3-(1H-imidazol-1-yl)propanoate (35).** Compound 35 was synthesised without triehtylamine and using dichloromethane (5 ml/mmol) as solvent. Light yellow oil in quantitative yield. ^1^H NMR (400 MHz, CDCl_3_) δ 7.51 (d, *J* = 8.0 Hz, 1H), 7.01 (d, *J* = 12.1 Hz, 1H), 6.91 (s, 1H), 4.59 (ddd, *J* = 14.4, 10.8, 5.8 Hz, 1H), 4.40 (ddd, *J* = 14.3, 7.4, 3.5 Hz, 1H), 4.28 − 4.08 (m, 6H), 3.43 − 3.26 (m, 1H), 1.47 − 1.28 (m, 6H), 1.29 − 1.13 (m, 3H).

**Ethyl 2-(diethoxyphosphoryl)-3-(1H-pyrazol-1-yl)propanoate (36).** Light yellow oil in 35% yield. ^1^H NMR (300 MHz, CDCl_3_) δ 7.50 (d, *J* = 1.5 Hz, 1H), 7.41 (d, *J* = 1.9 Hz, 1H), 6.18 (t, *J* = 2.1 Hz, 1H), 4.70 (ddd, *J* = 13.8, 10.5, 7.1 Hz, 1H), 4.57 (ddd, *J* = 13.8, 6.7, 4.1 Hz, 1H), 4.26 − 4.05 (m, 6H), 3.75 (ddd, *J* = 23.2, 10.5, 4.1 Hz, 1H), 1.35 (tdd, *J* = 7.0, 2.0, 0.4 Hz, 6H), 1.20 (t, *J* = 7.1 Hz, 3H).

**Ethyl 2-(diethoxyphosphoryl)-3-(1H-pyrrol-1-yl)propanoate (37).** Light yellow oil in 30% yield. ^1^H NMR (300 MHz, MeOD) δ 6.65 (t, *J* = 2.1 Hz, 2H), 6.01 (t, *J* = 2.1 Hz, 2H), 4.47 (ddd, *J* = 14.1, 10.5, 7.4 Hz, 1H), 4.32 (ddd, *J* = 14.1, 6.8, 4.2 Hz, 1H), 4.22 − 4.05 (m, 6H), 3.56 (ddd, *J* = 22.9, 10.5, 4.2 Hz, 1H), 1.43 − 1.26 (m, 6H), 1.19 (dd, *J* = 9.4, 4.9 Hz, 3H).

**Ethyl 2-(diethoxyphosphoryl)-3-(1H-1,2,4-triazol-1-yl)propanoate (38).** Light yellow oil in 86% yield. ^1^H NMR (400 MHz, MeOD) δ 8.47 (s, 1H), 7.98 (s, 1H), 4.86 − 4.77 (m, 1H), 4.61 (dd, *J* = 14.1, 7.4 Hz, 1H), 4.29 − 4.09 (m, 7H), 1.35 (dt, *J* = 7.0, 6.4 Hz, 6H), 1.21 (t, *J* = 7.1 Hz, 3H).

**General procedure for Wittig reaction (synthesis of compounds 3, 31, 39**–**42).** *t-*BuOK (1 M in THF, 1.2 eq) was added dropwise to a solution of the properly functionalised propanoate (1.1 equiv) in dry THF (0.9 ml/mmol) stirring at 0 °C under nitrogen atmosphere. After reacting for 30 min, the proper aldehyde (1 equiv) was added to the mixture, and the reaction was allowed to warm to room temperature. After complete consumption of the starting material according to TLC volatiles were evaporated. The residue was taken up with ethyl acetate (15 ml), that was washed with water (3 × 10 ml), brine, dried over anhydrous Na_2_SO_4_ and concentrated under reduced pressure to yield a residue that was purified by flash column chromatography. Yields, purification methods and other analytical data are reported below.

**Ethyl (E)-2-(4-methylbenzyl)-3-(pyridin-4-yl)acrylate (3).** Flash column chromatography eluting with ethyl acetate/petroleum ether (1:9 →  3:7) afforded the desired product as a yellowish oil in 42% yield. **E:** ^1^H NMR (300 MHz, CDCl3) δ 8.52 (d, *J* = 5.8 Hz, 2H), 8.02 (s, 1H), 7.49 − 7.31 (m, 5H), 7.15 (s, 2H), 4.24 (q, *J* = 7.1 Hz, 2H), 3.95 (s, 2H), 1.26 (dd, *J* = 12.0, 4.9 Hz, 3H).

**Ethyl (E)-3-phenyl-2-(pyridin-4-ylmethyl)acrylate (31).** Purification by flash column chromatography on silica gel using ethyl acetate/petroleum ether (2:8) allowed the isolation of the desired product in 40% yield as an orange oil. E and Z isomers were obtained. **E:** ^1^H NMR (300 MHz, CDCl_3_) δ 8.52 (d, *J* = 5.8 Hz, 2H), 8.02 (s, 1H), 7.49 − 7.31 (m, 5H), 7.15 (s, 2H), 4.24 (q, *J* = 7.1 Hz, 2H), 3.95 (s, 2H), 1.26 (dd, *J* = 12.0, 4.9 Hz, 3H).

**Ethyl (E)-2-((1H-imidazol-1-yl)methyl)-3-phenylacrylate (39).** Purification using methanol/dichloromethane (5:95) allowed the isolation of the product as a brown oil; 48% yield. **E:** ^1^H NMR (300 MHz, CDCl_3_) δ 8.03 (s, 1H), 7.40 (ddd, *J* = 32.4, 20.1, 7.5 Hz, 6H), 7.02 (s, 1H), 6.86 (s, 1H), 4.97 (s, 2H), 4.26 (q, *J* = 7.1 Hz, 2H), 1.30 (t, *J* = 7.1 Hz, 3H).

**Ethyl (E)-2-((1H-pyrazol-1-yl)methyl)-3-phenylacrylate (40).** Purification using ethyl acetate/petroleum ether (5:9 5 → 2:8) allowed the isolation of the product as a colourless oil in 31% yield. **E:** ^1^H NMR (300 MHz, CDCl_3_) δ 8.03 (s, 1H), 7.67 (dd, *J* = 7.7, 1.3 Hz, 2H), 7.54 (dd, *J* = 8.7, 2.0 Hz, 2H), 7.47 − 7.36 (m, 3H), 6.32 − 6.20 (m, 1H), 5.18 (s, 2H), 4.25 (q, *J* = 7.1 Hz, 2H), 1.30 (t, *J* = 7.1 Hz, 3H).

**Ethyl (E)-2-((1H-pyrrol-1-yl)methyl)-3-phenylacrylate (41).** Purification using ethyl acetate/petroleum ether (1:99) allowed the isolation of the product as a colourless oil in 69% yield. It wasn't possible to separate E/Z isomers and they were used as a mixture in the next step. ^1^H NMR (300 MHz, CDCl_3_) δ 7.98 (s, 1H), 7.38 (dd, *J* = 7.5, 2.2 Hz, 5H), δ 7.32 − 7.18 (m, 5H), 6.72 (t, *J* = 2.1 Hz, 2H), 6.65 (t, *J* = 2.1 Hz, 2H), 6.57 (s, 1H), 6.12 (t, *J* = 2.2 Hz, 4H), 4.94 (s, 2H), 4.84 (d, *J* = 1.5 Hz, 2H), 4.25 (q, *J* = 7.1 Hz, 2H), 4.09 (q, *J* = 7.1 Hz, 2H), 1.29 (t, *J* = 7.1 Hz, 3H),1.06 (t, *J* = 7.1 Hz, 3H).

**Ethyl (E)-2-((1H-1,2,4-triazol-1-yl)methyl)-3-phenylacrylate (42).** Purification using ethyl acetate/petroleum ether (2:8 →  1:1) allowed the isolation of the product as a white powder in 44% yield. **E:** ^1^H NMR (300 MHz, CDCl_3_) δ 8.23 (s, 1H), 8.06 (s, 1H), 7.97 (s, 1H), 7.77 − 7.62 (m, 2H), 7.57 − 7.34 (m, 3H), 5.20 (s, 2H), 4.26 (q, *J* = 7.1 Hz, 2H), 1.31 (t, *J* = 7.1 Hz, 3H).

**General procedure for Corey-Chaykovsky reaction (synthesis of compounds 4, 32, 43**–**46).** Trimethylsufoxonium iodide (1.2 equiv) was added to a suspension of NaH (1.2 equiv) in dry DMSO (5 ml/mmol) stirred under nitrogen atmosphere, and the reaction mixture was stirred at room temperature for 1 h. After cooling to 0 °C, a solution of the desired acrylate intermediate (1 equiv) in dry DMSO (2 ml/mmol) was added dropwise over 20 min, and the resulting mixture was allowed to stir at room temperature overnight. Water (80 ml) was added and the resulting solution was extracted with ethyl acetate (3 × 30 ml). The organic phases were separated, washed with brine, dried over anhydrous Na_2_SO_4_, filtered and concentrated under reduced pressure to give a residue that is purified by flash column chromatography to yield the desired intermediate. Yields, purification methods and other analytical data are reported below.

**Ethyl-1-(4-methylbenzyl)-2-(pyridin-4-yl)cyclopropane-1-carboxylate (4)**. Purification by column chromatography eluting ethyl acetate/petroleum ether (1:9 →  3:7) afforded the product as a yellowish oil in 72% yield. ^1^H NMR (300 MHz, CDCl_3_) δ: 7.17 − 7.14 (m, 4H); 7.05 − 6.99 (m, 4H); 4.19 − 4.12 (m, 2H); 3.12 (d, *J* = 15.54, 1H); 2.82 (t, *J* = 8.1, 1H); 2.30 (s, 3H); 2.07 (d, *J* = 15.69, 1H); 1.93 − 1.88 (m, 1H); 1.46 (t, *J* = 6.72, 1H); 1.24 (t, *J* = 9.03, 3H).

**Ethyl 2-phenyl-1-(pyridin-4-ylmethyl)cyclopropane-1-carboxylate (32).** Purification by flash column chromatograpy using ethyl acetate/petroleum ether (2:8 →  4:6) allowed the isolation of the desired compound in 48% yield as a yellow oil. ^1^H NMR (300 MHz, CDCl_3_) δ 8.42 (s, 2H), 7.41 − 7.14 (m, 5H), 7.08 (d, *J* = 4.5 Hz, 2H), 4.13 (qd, *J* = 7.1, 4.0 Hz, 2H), 3.10 (d, *J* = 16.1 Hz, 1H), 2.90 (dd, *J* = 20.2, 12.7 Hz, 1H), 2.11 (d, *J* = 16.1 Hz, 1H), 1.92 (ddd, *J* = 9.2, 5.0, 1.2 Hz, 1H), 1.42 (dd, *J* = 7.2, 5.0 Hz, 1H), 1.17 (t, *J* = 7.1 Hz, 3H).

**Ethyl 1-((1H-imidazol-1-yl)methyl)-2-phenylcyclopropane-1-carboxylate (43).** Purification using methanol/dichloromethane (1:99 → 5:95) afforded the product as a brown oil in 64% yield. ^1^H NMR (400 MHz, CDCl_3_) δ 7.34 (ddd, *J* = 14.6, 7.7, 6.2 Hz, 3H), 7.25 − 7.16 (m, 3H), 6.93 (s, 1H), 6.73 (s, 1H), 4.34 (dd, *J* = 14.9, 0.6 Hz, 1H), 4.21 (qd, *J* = 7.1, 2.7 Hz, 2H), 3.45 (d, *J* = 14.9 Hz, 1H), 3.13 − 3.01 (m, 1H), 1.82 (ddd, *J* = 9.2, 5.3, 1.0 Hz, 1H), 1.50 (dd, *J* = 7.4, 5.4 Hz, 1H), 1.28 (t, *J* = 7.1 Hz, 3H).

**Ethyl 1-((1H-pyrazol-1-yl)methyl)-2-phenylcyclopropane-1-carboxylate (44).** Purification using ethyl acetate/petroleum ether (5:9 5 → 1:9) afforded the product as a colourless oil in 58% yield. ^1^H NMR (300 MHz, CDCl_3_) δ 7.46 (dd, *J* = 6.3, 2.0 Hz, 2H), 7.41–7.27 (m, 5H), 6.18 (t, *J* = 2.1 Hz, 1H), 4.70 (d, *J* = 14.7 Hz, 1H), 4.31 − 4.11 (m, 2H), 3.43 (d, *J* = 14.8 Hz, 1H), 2.99 (t, *J* = 8.4 Hz, 1H), 1.91 − 1.77 (m, 2H), 1.27 (t, *J* = 7.1 Hz, 3H).

**Ethyl 1-((1H-pyrrol-1-yl)methyl)-2-phenylcyclopropane-1-carboxylate (45).** Purification using ethyl acetate/petroleum ether (0.8:99.2) afforded the product as a slightly yellow solid in 33% yield. ^1^H NMR (300 MHz, CDCl_3_) δ 7.31 (ddd, *J* = 18.0, 14.2, 6.7 Hz, 5H), 6.53 (t, *J* = 1.9 Hz, 2H), 6.06 (t, *J* = 1.9 Hz, 2H), 4.48 (d, *J* = 14.9 Hz, 1H), 4.32 − 4.07 (m, 2H), 3.21 (d, *J* = 14.9 Hz, 1H), 3.00 (t, *J* = 8.3 Hz, 1H), 1.83 (dd, *J* = 8.7, 5.4 Hz, 1H), 1.50 (dd, *J* = 7.3, 5.4 Hz, 1H), 1.29 (t, *J* = 7.2 Hz, 3H).

**Ethyl 1-((1H-1,2,4-triazol-1-yl)methyl)-2-phenylcyclopropane-1-carboxylate (46).** Purification using a gradient of ethyl acetate in petroleum ether afforded the product as a yellow oil in 38% yield. ^1^H NMR (300 MHz, CDCl_3_) δ 8.09 (d, *J* = 13.9 Hz, 1H), 7.86 (s, 1H), 7.42 − 7.25 (m, 5H), 4.71 (d, *J* = 14.7 Hz, 1H), 4.29 − 4.14 (m, 2H), 3.46 (dd, *J* = 14.4, 2.1 Hz, 1H), 3.17 − 3.01 (m, 1H), 1.86 (ddt, *J* = 9.2, 5.4, 3.3 Hz, 2H), 1.28 (t, *J* = 7.1 Hz, 3H).

### General procedure for the hydrolysis of the esters (synthesis of compounds 5, 8, 10, 12, 15, 33, 47–50):

LiOH (4 equiv) was added to a solution of the ester (1 equiv) in THF/MeOH/H_2_O (3:1:1, 1 ml/mmol) and the reaction mixture was heated in a microwave reactor set with the following parameters: 100 °C, 10 min, 300 W, 250 psi. After concentration of the mixture, 2 N HCl aqueous solution (10 ml) was added and the resulting slurry was extracted with ethyl acetate (3 × 20 ml). The collected organic phases were washed with brine, dried over anhydrous Na_2_SO_4_, filtered and concentrated under reduced pressure to give a crude material that is purified by flash column chromatography. Yields, purification methods and other analytical data are reported below.

**1-(4-methylbenzyl)-2-(pyridin-4-yl)cyclopropane-1-carboxylic acid (5).** Purification by column chromatography eluting with ethyl acetate/petroleum ether (1:9 →  3:7) afforded the desired product as a yellowish powder in 64% yield. ^1^H NMR (300 MHz, CDCl_3_) δ: 7.23 − 7.17 (m, 4H); 7.11 − 7.05 (m, 4H); 3.23 (d, *J* = 15.54, 1H); 2.87 (t, *J* = 8.1, 1H); 2.32 (s, 3H); 2.11 (d, *J* = 15.69, 1H); 1.96 − 1.92 (m, 1H); 1.53 (t, *J* = 6.72, 1H).^13^C NMR (100.6 MHz, CDCl_3_) δ: 183.6; 142.3; 127.4; 126.7; 126.4; 125.2; 124.2; 122.0; 35.0; 33.6; 30.7; 18.7; 11.4. MS (ESI): m/z: 268.5 [M + H]

**1-(4-methylbenzyl)-2-(4-nitrophenyl)cyclopropane-1-carboxylic acid (8).** The product was obtained as a pale yellow oil in 47% yield after purification with methanol/dichloromethane (1:99). ^1^H NMR (400 MHz, CDCl_3_) δ 8.20 (dd, *J* = 20.6, 11.5 Hz, 2H), 7.45 (d, *J* = 11.1 Hz, 2H), 7.14 − 6.98 (m, 4H), 3.18 (d, *J* = 15.7 Hz, 1H), 3.05 (t, *J* = 8.1 Hz, 1H), 2.34 (d, *J* = 9.4 Hz, 3H), 2.04 (dd, *J* = 10.8, 7.2 Hz, 2H), 1.63 − 1.53 (m, 1H). ^13 ^C NMR (101 MHz, CDCl_3_) δ 180.1, 147.3, 144.3, 136.1, 136.1, 130.3, 129.2, 128.6, 123.9, 33.2, 32.9, 31.6, 21.2, 19.5. HRMS (ESI): calculated for C_18_H_16_O_4_N [M-H] 310.10738 found 310.10849.

**2-(4-((isopropylamino)methyl)phenyl)-1-(4-methylbenzyl)cyclopropane-1-carboxylic acid (10).** Purification by flash chromatography on silica gel methanol/dichloromethane (8:9 2 → 1:9) afforded the product as using white powder in 55% yield. ^1^H NMR (400 MHz, MeOD) δ 7.48 (d, *J* = 8.1 Hz, 2H), 7.38 (d, *J* = 8.1 Hz, 2H), 7.00 (q, *J* = 8.1 Hz, 4H), 4.20 (s, 2H), 3.43 (dt, *J* = 13.1, 6.5 Hz, 1H), 3.37 (s, 1H), 3.05 (d, *J* = 15.5 Hz, 1H), 2.93 − 2.79 (m, 1H), 2.25 (s, 3H), 2.04 (d, *J* = 15.6 Hz, 1H), 1.78 (dd, *J* = 8.8, 4.9 Hz, 1H), 1.40 (d, *J* = 6.5 Hz, 7H). ^13 ^C NMR (101 MHz, MeOD) δ 180.6, 139.2, 137.3, 134.7, 129.7, 129.7, 129.4, 128.1, 128.1, 50.4, 33.0, 31.6, 31.2, 19.5, 17.8, 16.6. HRMS (ESI): calculated for C_22_H_27_O_2_N [M-H] 336.19581 found 336.19809.

**2-(4-aminophenyl)-1-(4-methylbenzyl)cyclopropane-1-carboxylic acid (12).** The target compound was obtained as an orange semi-solid compound in 40% yield after purification with methanol/dichloromethane (2:98). ^1^H NMR (400 MHz, CDCl_3_) δ 7.10 − 7.00 (m, 6H), 6.68 (d, *J* = 8.4 Hz, 2H), 3.20 (d, *J* = 15.7 Hz, 1H), 2.90 − 2.78 (m, 1H), 2.30 (s, 3H), 1.88 (dd, *J* = 14.5, 9.0 Hz, 2H), 1.36 (dd, *J* = 7.2, 5.0 Hz, 1H). ^13 ^C NMR (101 MHz, CDCl_3_) δ 180.8, 145.2, 137.1, 135.3, 130.2, 128.8, 128.5, 126.2, 115.2, 33.9, 32.6 30.6, 21.0, 18.5. HRMS (ESI): calculated for C_18_H_19_O_2_N [M-H] 280.13321 found 280.13467.

**1-(4-methylbenzyl)-2-phenylcyclopropane-1-carboxylic acid (15).** Purification by flash chromatography on silica gel using methanol/dichloromethane (1:99) afforded the product as a yellow powder in 80% yield. ^1^H NMR (400 MHz, CDCl_3_) δ 7.46 − 7.21 (m, 5H), 7.06 (d, *J* = 13.0 Hz, 4H), 3.20 (d, *J* = 15.8 Hz, 1H), 2.97 (t, *J* = 7.9 Hz, 1H), 2.30 (s, 3H), 1.91 (d, *J* = 16.4 Hz, 2H), 1.47 (s, 1H).

**2-phenyl-1-(pyridin-4-ylmethyl)cyclopropane-1-carboxylic acid (33).** The ethyl acetate organic phase was discarded and the combined organic phases obtained with the chloroform/isopropanol mixture were dried over anhydrous sodium sulphate, filtered and concentrated under reduced pressure to afford 110 mg of the desired compound as a brown solid (25% yield). ^1^H NMR (400 MHz, MeOD) δ 8.60 (d, *J* = 6.5 Hz, 2H), 7.84 (d, *J* = 6.4 Hz, 2H), 7.36 − 7.19 (m, 5H), 3.24 − 3.14 (m, 1H), 3.09 (dd, *J* = 9.2, 7.4 Hz, 1H), 2.75 (dd, *J* = 18.3, 10.7 Hz, 1H), 2.00 − 1.89 (m, 1H), 1.78 (dd, *J* = 7.3, 5.1 Hz, 1H). ^13 ^C NMR (101 MHz, MeOD) δ 177.0, 164.9, 141.9, 137.2, 130.2, 129.5, 128.5, 35.9, 32.9, 30.6, 19.2. HRMS (ESI): calculated for C_16_H_14_O_2_N [M-H] 252.10191 found 252.10334.

**1-((1H-imidazol-1-yl)methyl)-2-phenylcyclopropane-1-carboxylic acid (47).** Extractions with chloroform/isopropanol of the water phase allowed the isolation of the product as a light brown solid in 65% yield. ^1^H NMR (400 MHz, MeOD) δ 8.41 (s, 1H), 7.47 − 7.17 (m, 7H), 4.37 (d, *J* = 14.9 Hz, 1H), 4.06 (d, *J* = 14.9 Hz, 1H), 3.14 (dd, *J* = 10.0, 6.7 Hz, 1H), 1.96 (dd, *J* = 7.5, 5.4 Hz, 1H), 1.89 − 1.78 (m, 1H). ^13 ^C NMR (101 MHz, MeOD) δ 175.4, 137.0, 135.9, 130.0, 129.9, 128.8, 123.5, 120.3, 50.5, 33.0, 31.0, 19.0. HRMS (ESI): calculated for C_14_H_13_O_2_N_2_ [M-H] 241.09715 found 241.09817.

**1-((1H-pyrazol-1-yl)methyl)-2-phenylcyclopropane-1-carboxylic acid (48).** Purification by flash column chromatography using methanol/dichloromethane (2.5:97.5) with 0.01% formic acid allowed the isolation of the desired product as a light brown powder in 89% yield. ^1^H NMR (400 MHz, CDCl_3_) δ 7.51 (s, 1H), 7.40 − 7.21 (m, 5H), 7.17 (s, 1H), 6.16 (s, 1H), 4.53 (d, *J* = 15.0 Hz, 1H), 3.68 (d, *J* = 15.1 Hz, 1H), 3.10 (t, *J* = 8.3 Hz, 1H), 1.92 (dd, *J* = 8.8, 5.4 Hz, 1H), 1.85 − 1.74 (m, 1H). ^13 ^C NMR (101 MHz, CDCl_3_) δ 177.8, 139.0, 135.1, 130.6, 129.2, 128.8, 127.7, 105.4, 50.5, 33.8, 31.0, 18.1. HRMS (ESI): calculated for C_14_H_13_O_2_N_2_ [M-H] 241.09715 found 241.09776.

**1-((1H-pyrrol-1-yl)methyl)-2-phenylcyclopropane-1-carboxylic acid (49).** Purification by flash column chromatography using methanol/dichloromethane (1:99) afforded the product as beige powder in 63% yield. ^1^H NMR (300 MHz, CDCl_3_) δ 7.44 − 7.21 (m, 5H), 6.57 (s, 2H), 6.08 (s, 2H), 4.51 (d, *J* = 15.1 Hz, 1H), 3.15 (dd, *J* = 17.8, 11.7 Hz, 2H), 1.92 (dd, *J* = 8.7, 5.6 Hz, 1H), 1.71 − 1.52 (m, 1H). ^13 ^C NMR (75 MHz, CDCl_3_) δ 179.6, 134.7, 128.8, 128.5, 127.4, 120.8, 107.6, 47.0, 33.9, 31.1, 17.5. HRMS (ESI): calculated for C_15_H_14_O_2_N [M-H] 240.10191 found 240.10257.

**1-((1H-1,2,4-triazol-1-yl)methyl)-2-phenylcyclopropane-1-carboxylic acid (50).** Purification by flash column chromatography using methanol/dichloromethane (1:99) with 0.01% formic acid afforded the pure product as a white solid in 53% yield. ^1^H NMR (400 MHz, CDCl_3_) δ 8.49 (s, 1H), 7.95 (s, 1H), 7.46 − 7.21 (m, 5H), 4.80 (d, *J* = 14.7 Hz, 1H), 3.48 (d, *J* = 14.7 Hz, 1H), 3.19 (t, *J* = 8.3 Hz, 1H), 1.92 (dd, *J* = 10.6, 5.3 Hz, 2H).^13^C NMR (101 MHz, CDCl_3_) δ 176.0, 149.6, 143.9, 134.8, 128.9, 128.6, 127.5, 49.2, 32.9, 30.1, 17.4. HRMS (ESI): calculated for C_13_H_12_O_2_N_3_ [M-H] 242.09240 found 242.09300.

**Diethyl (1-cyano-2-phenylethyl)phosphonate (27).** Diethyl cyanomethylphosphonate **26** (2.43 ml, 1.5 mmol) was added under nitrogen atmosphere to a suspension of NaH (60% dispersion in mineral oil, 900 mg, 2.25 mmol) in dry dimethoxyethane (15 ml) kept at 0 °C. After stirring 2 h at room temperature, benzyl bromide (2.14 ml, 1.8 mmol) was added and the reaction was heated at 60 °C for 2 h. Water (10 ml) was carefully added and the resulting solution was extracted with ethyl acetate (3 × 10 ml). The organic phases were washed with brine, dried over anhydrous Na_2_SO_4_, filtered and concentrated under reduced pressure to give a crude material that is purified by flash column chromatography using 10% ethyl acetate in petroleum ether as the eluent. The product was obtained as an oil in 90% yield. ^1^H NMR (300 MHz, CDCl_3_) δ 7.39 − 7.21 (m, 5H), 4.37 − 4.15 (m, 4H), 3.21 (ddt, *J* = 19.2, 11.1, 5.9 Hz, 3H), 1.38 (td, *J* = 7.1, 2.9 Hz, 6H).

***(1R,2S)*-1-benzyl-2-phenylcyclopropanecarbonitrile (28).** *n-*BuLi (5.4 ml, 13.48 mmol) was added under nitrogen atmosphere dropwise to a solution of diethyl (1-cyano-2-phenylethyl)phosphonate **27** (3.6 g, 13.48 mmol) in dry dimethoxyethane (15 ml) at room temperature. After stirring for 30 min, styrene oxide (1.03 ml, 9 mmol) was added in one portion and the reaction mixture was stirred at 90 °C overnight. After consumption of the starting material, saturated NH_4_Cl aq. solution (10 ml) was added and the resulting solution was extracted with ethyl acetate (3 × 100 ml). The organic phases were washed with brine, dried over anhydrous Na_2_SO_4_, filtered and concentrated under reduced pressure to give a crude material that is purified by flash column chromatography using 5% ethyl acetate in petroleum ether afforded the title compound as a white solid in 25% yield. ^1^H NMR (300 MHz, CDCl_3_) δ 7.46 − 7.25 (m, 8H), 7.09 (dd, *J* = 7.6, 1.6 Hz, 2H), 3.10 − 2.94 (m, 1H), 2.74 (d, *J* = 15.0 Hz, 1H), 2.22 (d, *J* = 15.0 Hz, 1H), 1.74 (ddd, *J* = 9.3, 5.9, 0.7 Hz, 1H), 1.49 (ddd, *J* = 11.6, 5.5, 3.9 Hz, 1H). ^13^ C NMR (75 MHz, CDCl_3_) δ 137.0, 133.9, 129.2, 129.0, 128.8, 128.7, 128.6, 127.8, 127.2, 123.6, 35.4, 30.6, 17.3. HRMS (ESI): calculated for C_17_H_15_N [M + H] 234.1204 found 234.1283.

***(1R,2S)*-5-(1-benzyl-2-phenylcyclopropyl)-1H-tetrazole (29).** Sodium azide (78 mg, 1.56 mmol) and triethylamine (167 µL, 1.2 mmol) were added to a solution of 1-benzyl-2-phenylcyclopropanecarbonitrile **28** (90 mg, 0.39 mmol) in DMF (3 ml) at room temperature. The reaction mixture was stirred under nitrogen atmosphere at 130 °C for 16 h until TLC monitoring revealed complete consumption of the starting material. 1 N HCl aq. solution (10 ml) was added and the resulting solution was extracted with ethyl acetate (3 × 10 ml). The organic phases were washed with brine, dried over anhydrous Na_2_SO_4_, filtered and concentrated under reduced pressure to give a crude material that is purified by flash column chromatography using 33% ethyl acetate in petroleum ether. The product was obtained as an oil in 46% yield. ^1^H NMR (300 MHz, CDCl_3_) δ 7.92 (s, 1H), 7.46 − 7.09 (m, 10H), 3.19 (d, *J* = 16.3 Hz, 1H), 2.95 (dt, *J* = 22.9, 11.4 Hz, 1H), 2.49 (d, *J* = 16.3 Hz, 1H), 2.01 (ddd, *J* = 9.1, 5.3, 1.2 Hz, 1H), 1.72 (dd, *J* = 7.0, 5.3 Hz, 1H). ^13 ^C NMR (101 MHz, CDCl_3_) δ 162.7, 139.1, 136.9, 129.3, 128.8, 128.5, 127.0, 126.7, 35.7, 32.4, 25.0, 19.1. MS (ESI): m/z: 275 [M-H].

## Results

### Rationale for modifications

**Figure 2.**
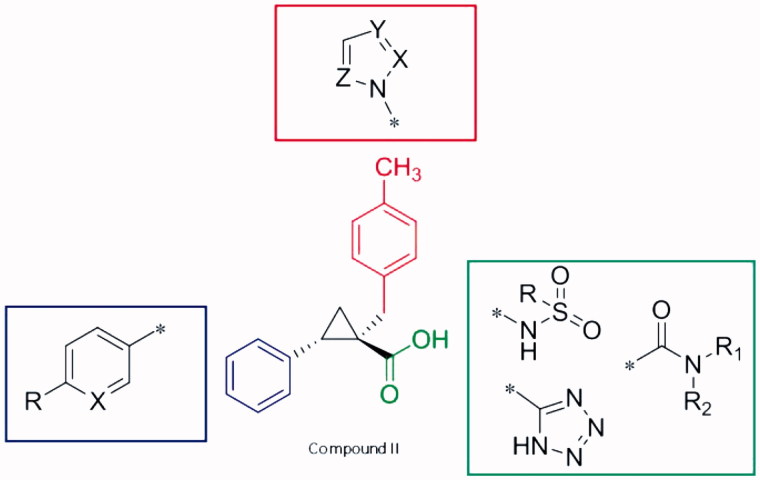
Proposed analogues of OASS inhibitor **II**.

In our previous works, the structure of a poorly bioavailable peptide inhibitor was first simplified to obtain a substituted cyclopropane carboxylic acid, the further optimisation of which led to compound **II**, a nanomolar binder of OASS-A^13^. The structure activity relationships (SAR) for this series of 2-phenylcyclopropane carboxylic acid derivatives highlighted the fact that a carboxylic moiety and an aromatic substituent kept at a suitable distance are crucial for the activity^27^. The cyclopropane ring, that maintains the two moieties in the *trans* configuration, proved to be a valuable spacer. An additional substitution at the C1 of the cyclopropane strongly affects both the potency of the compound and its selectivity toward both *St*OASS-A and *St*OASS-B isoforms. Interestingly, stereochemistry proved to be extremely important in conferring high potency, as the *2S* enantiomer resulted more potent than the *2 R* counterpart by a 1000-fold average. Based on these preliminary hints, in this work we have further investigated the chemical space around compound **II** with the aim of maintaining high inhibitory potency of the enzyme while improving the bacterial cell penetration. The aromatic ring attached at the C2 of the cyclopropane, the carboxylic acid, and the benzylic moiety attached at the C1, were considered as key characteristics for the activity. Taking this into account, modifications of the molecule at three different parts were proposed (Figure 2).

Combination of funnel metadynamics with Saturation Transfer Difference (STD)-NMR technique has shown that the phenyl ring at the C2 can be variously substituted^28^. We have already demonstrated that bulky groups such as aromatic and heteroaromatic rings are allowed due to the presence of a hydrophobic pocket at the target binding site. Since a higher amphiphilic moiety may favour permeability^16^ ^,^ ^17^, amine groups were introduced at the *para* position of the C2 phenyl ring. Also, the phenyl ring was substituted by a pyridine, to improve the overall drug-likeness of the molecule.

To investigate whether the carboxylic acid functionality might be modified, we replaced it with isosteric groups. Among the set of possible substituents, sulphonamides, a tetrazole ring and amides were initially investigated. While the sulphonamide group is a nonplanar isoster of the carboxylic acid, the tetrazole is planar and presents a similar acidity. On a similar vein, substituted amides were prepared because it is well known that the nature of the substituents might affect the selectivity of action toward different bacterial strains, either Gram-positive or Gram-negative^29^ ^,^ ^30^. For instance, in the case of sulphonamide drugs, potency and selectivity are modulated by the substituent at the amidic nitrogen^31^. Finally, we investigated the benzyl group attached at the C1 of the cyclopropane ring, with the aim to evaluate its substitution with heteroaromatic structures like pyridine and five-terms heteroaromatic rings, leading to molecules characterised by a lower lipophilicity.

## Discussion

Medicinal chemistry rationales were used to modify the structure of cyclopropane carboxylic acids as inhibitors of OASS in a Gram-negative bacterium. A total of 20 compounds were designed and synthesised in this study to evaluate the effect of the chemical modifications to both enzymatic and antibacterial activities, leading to additional SAR information. The cyclopropane motif was kept since it contributes to the favourable positioning of the substituents in OASS pocket, and, moreover, it provides rigidity to the molecule. The new set of molecules was then biochemically evaluated on both isoforms of the recombinant OASS enzyme from *Salmonella* to determine their potency (Table 1). For most compounds the potency was assessed by fluorometric titration, exploiting the increase in the fluorescence emission of the cofactor upon binding to the active site. Compounds with substituents different from carboxylate were unable to elicit the fluorescence changes and for this reason their potency was measured by activity assays.

**Table 1.** Inhibitory potency of compounds **5**, **8**, **10**, **12**, **16–22**, **24, 25, 28, 29, 33, 47–50**.

Compound	OASS-A		OASS-B	
	*K*_d_ (μM)	IC_50_ (μM)	*K*_d_ (μM)	IC_50_ (μM)
**5**	0.4 ± 0.1	ND	6.1 ± 0.5	ND
**8**	61 ± 6	ND	145 ± 2	ND
**10**	155 ± 5	ND	¥	NDS
**12**	1.4 ± 0.3	ND	5.7 ± 0.7	ND
**16**	¥	17 ± 2	¥	137 ± 10
**17**	¥	>100	¥	>100
**18**	¥	>100	¥	>100
**19**	¥	>100	¥	>100
**20**	¥	>100	¥	>100
**21**	¥	>100	¥	>100
**22**	¥	>100	¥	>100
**24**	¥	PI (45%)^a^	¥	PI (50%)^a^
**25**	¥	PI (39%)^a^	¥	PI (35%)^a^
**28**	¥	>250	¥	NDS
**29**	90 ± 14	ND	NDS	ND
**33**	24 ± 2	ND	275 ± 42	ND
**47**	7.6 ± 0.5	ND	397 ± 42	ND
**48**	1.5 ± 0.2	ND	96 ± 7	ND
**49**	0.5 ± 0.1	ND	22 ± 2	ND
**50**	18 ± 5	ND	250 ± 14	ND
**II**	0.03 ± 0.01	ND	0.5 ± 0.1	ND

ND: not determined; NDS: not determined due to solubility issues; PI: partial inhibitor.

a values in brackets are the percent inhibition measured at saturation of inhibitor or at the highest inhibitor concentration allowed by solubility.

¥: *K*_d_ could not be calculated since compound binding does not cause any change in fluorescence emission.

**Scheme 1.**
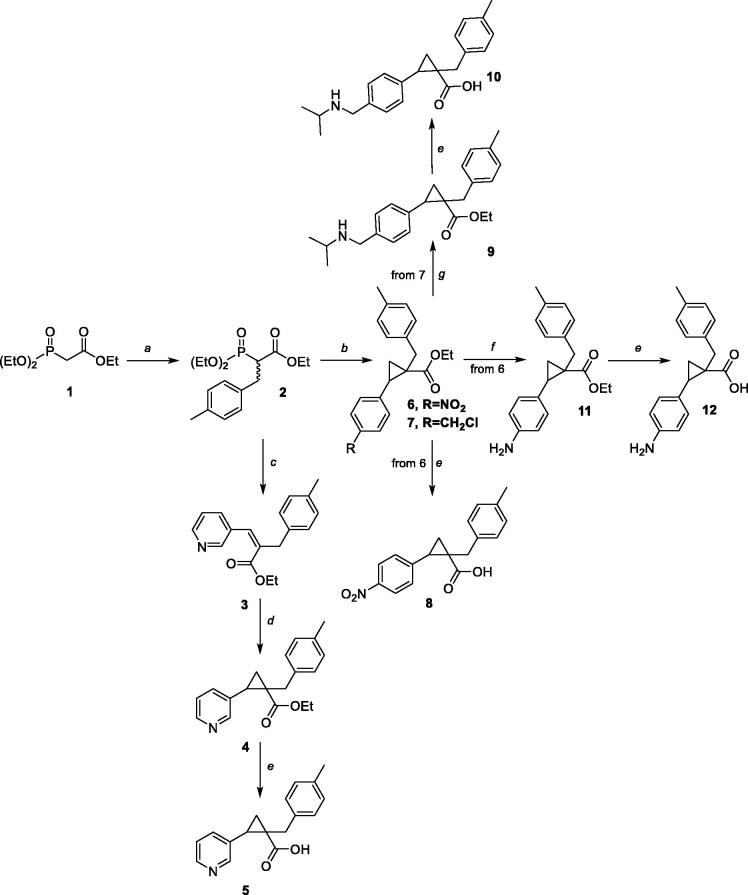
Synthetic routes to compounds **5**, **8**, **10** and **12**. Reagents and Conditions**:** (a) NaH, DME, 4-MeBnBr, RT-60 °C; 63%; (b) *n*-BuLi, DME, (R)-phenyloxirane, RT- 90 °C; 11%–20% (c) *t-*BuOK, THF, 0 °C–RT; 86%; (d) (CH_3_)_3_SOI, NaH, DMSO; 0 °C–RT; 54%; (e) LiOH, THF/MeOH/H_2_O, 100 °C; 50–60%; (f) TES, Pd/C, MeOH, RT; 100%; (g) NH_2_CH(CH_3_)_2_, RT; 55%;

**Scheme 2.**
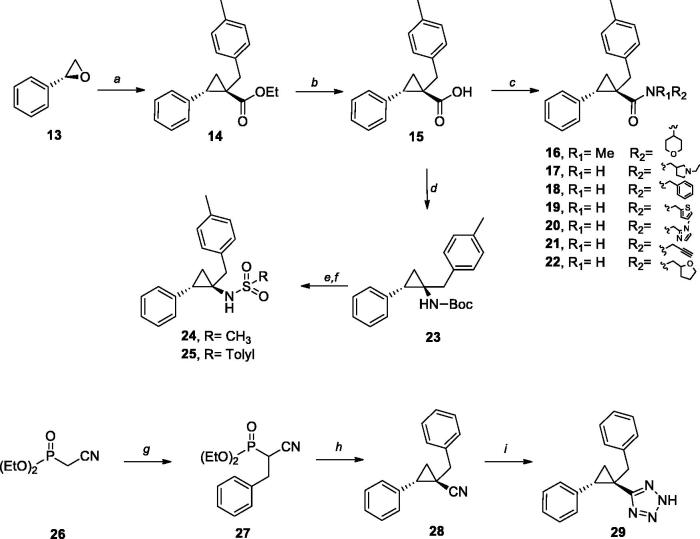
Synthetic routes to compounds **16–22**, **24**, **25**, **28** and **29**. Reagents and Conditions**:** (a) *n*-BuLi, DME, ethyl 2-(diethoxyphosphoryl)-3-(p-tolyl)propanoate (2), RT-90 °C; 63%; (b) LiOH, THF/MeOH/H_2_O, 100 °C; 87%; (c) HOBt, DIPEA, EDC.HCl, DMF, R_1_R_2_NH; 34–60%; (d) TEA, DPPA, *t*-BuOH, RT, 50%; (e) TFA, DCM, RT;100%; (f) TEA, RSO_2_Cl; −15 °C; 50–61%. (g) NaH, DME, BnBr, RT-60 °C, 90%; (h) *n*-BuLi, DME, Phoxirane, RT-90 °C, 25%; (i) NaN_3_, TEA, DMFA, 130 °C, 46%.

**Figure 3.**
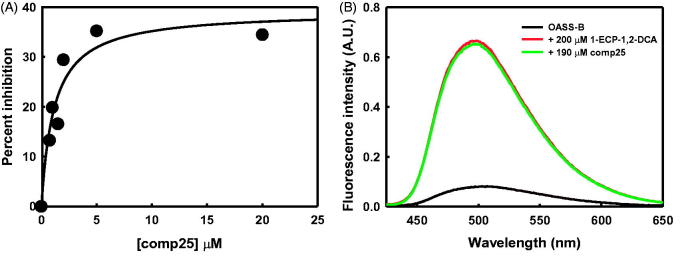
Interaction of compound 25 with OASS-B. Panel (A) Dependence of the percent inhibition of OASS-B catalytic activity on compound 25 concentration. Activity assays were carried out in the presence of 0.6 mM bisulphide and 1 mM OAS at 25 °C. Line through data points is the fitting to a hyperbolic function, drawn to guide the eye. Panel (B) Competitive binding assay. Fluorescence emission spectra upon excitation at 412 nm were collected in the absence and presence of 200 µM 1-ethylcyclopropane-1,2-dicarboxylic acid (1-ECP-1,2-DCA). The increase in the fluorescence emission indicates the formation of a specific complex with OASS-B. Addition of 190 µM compound 25 does not change the emission spectrum. Spectra were collected in 100 mM Hepes buffer, 1% DMSO, pH 7, 20 °C.

Regarding the C2 phenyl ring (Scheme 1), small electron donor groups (EDGs) such as the amino moiety (compound **12**, *K*_dOASS-A_ = 1.4 μM), when not substituted, allowed to maintain good binding properties, whereas bulkier groups such as the nitro and a substituted alkylamine (compound **8**, *K*_d__OASS-A_ = 61 μM; compound **10**, *K*_dOASS-A_ = 155 μM) weakened the interaction of the molecule with the active pocket, thus decreasing the affinity. It is possible to conclude that expansion of the series towards the *para* position of the phenyl ring, unless carried out with small groups, is detrimental for the activity of the compounds. To increase the polarity of the molecule, substitution of the C2 phenyl ring with a pyridine showed to be a more fruitful strategy, with high affinity toward both isoforms (compound **5**, K_dOASS-A_ = 0.4 μM, *K*_dOASS-B_ = 6.1 μM). Even though the carboxylic acid group is essential for the binding to OASS^13^ ^,^ ^27^ ^,^ ^28^, its scarce diffusion across biological membranes is a well-known issue^32^ ^,^ ^33^. Therefore, to preserve the key interactions with the target, we carried out the replacement of the carboxylic acid by several isosters (Scheme 2). Substitution with a sulphonamide led to molecules unable to elicit significant changes in the fluorescence emission of the cofactor, potentially indicating that the compounds did not bind to the active site of the enzyme. This prompted us to further investigate the mechanism of inhibition of these molecules, since the residual activity at saturating concentrations is significant (about 40 − 50%, Figure 3, panel A). The molecules are poorly soluble in the buffer used for activity assays, compound 24 having a solubility limit of about 250 μM and compound 25 of about 50 μM. This hampers the accurate determination of the inhibition mechanism due to limitations to the range of inhibitor concentration that can be explored. However, since partial inhibition is a typical feature of allosteric inhibitors, we used a method developed by our group^24^ to assess if compound **25** is able to displace a low affinity active site binder, 1-ethylcyclopropane-1,2-dicarboxylic acid, that forms a highly fluorescent complex with OASS. Compound **25** is unable to displace the compound up to an equimolar concentration of about 200 μM. This is a strong indication that compound **25** is not able to compete for binding to the active site with 1-ethylcyclopropane-1,2-dicarboxylic acid. Therefore, either the dissociation constant of compound **25** for OASS-B is much higher than 170 μM (the *K*_d_ of 1-ethylcyclopropane-1,2-dicarboxylic acid for OASS-B) or compound **25** does not bind to the active site of the enzyme (Figure 3, panel B). Unfortunately, replacement of the carboxylic acid by a tetrazole resulted in the decrease of the binding affinity (compound **29**, *K*_dOASS-A_ = 90 μM). Additional derivatization of the carboxylic acid group to deliver a small series of amides (compounds **16**–**22**) was attempted as well in order to gain further insights into the anchoring groups that can be tolerated by StOASS. Either unsubstituted or substituted amides, regardless of the moiety attached to the nitrogen atom, had in general a detrimental effect on the binding constant. Rather surprisingly, the good activity of the tertiary amide **16** (compound **16**, IC_50 OASS-A_ = 17.2 μM) represents an exception to the SAR information collected. Indeed, from the bulk of experimental data so far described, it can be speculated that carboxylic acid replacement is tolerable if at that position the presence of a sharable hydrogen is granted. By contrast, the lack of a hydrogen for H-bond formation in compound **16** makes difficult its collocation within the SAR. With the help of computational methods, further efforts on the study of the mechanism of binding for this molecule will help in the explanation of this odd result.

**Scheme 3.**
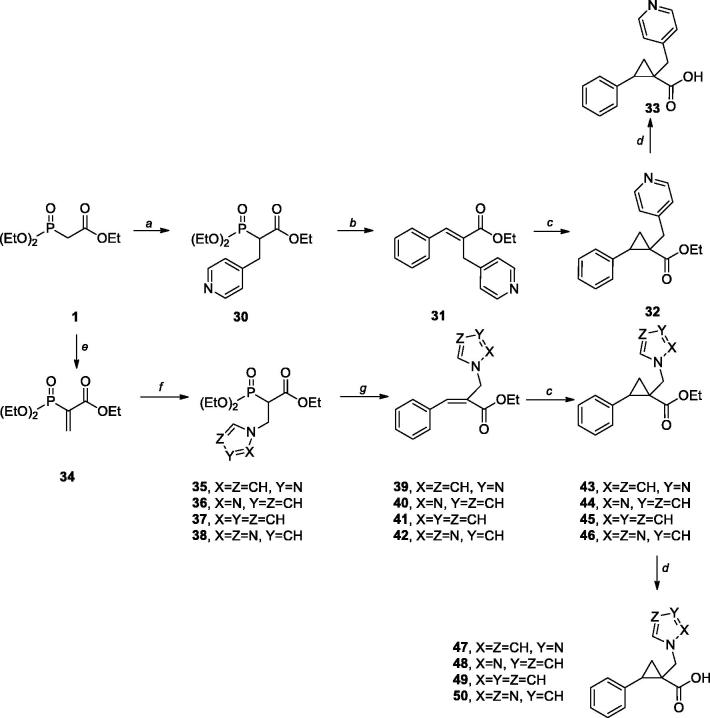
Synthetic routes to compounds **33**, **47–50**. Reagents and Conditions: (a) NaH, DMF/THF, 4-bromomethylpyridine, RT; 20%; (b) K_2_CO_3_, THF, benzaldehyde, RT-60 °C; 40% (c) NaH, (CH_3_)_3_SOI, DMSO, RT; 33–64%; (d) LiOH, THF/MeOH/H_2_O, 100 °C; 25–89%; (e) MeOH, piperidine, paraformaldehyde, *p*TSA, toluene, 100%; (f) proper heterocycle, CHCl_3_, Et_3_N, RT; (g) *t*-BuOK, THF, benzaldehyde, RT; 11–38%;

Finally, the benzylic moiety at the C1 position showed to be the most suitable for chemical intervention (Scheme 3). Its substitution with smaller size heterocycles, endowed with ameliorated drug-like characteristics, in all cases allowed to maintain good binding properties in the low micromolar or sub-micromolar range (compounds **33**, **47**–**50**, Table 1). To some extent, a correlation between polarity of the heterocycle and binding to the target enzyme seems to be present. In fact, higher polarity of compound somehow correlates with the decrease in binding affinity, with the pyrrole derivative (compound **49**, *K*_dOASS-A_ = 0.5 μM) being the most active of the series, and the triazole (compound **50**, *K*_dOASS-A_ = 18 μM) exhibiting the highest *K*_d_.

It is worthwhile to notice that one of the main hurdles in the development of small molecules inhibitors of OASS is the consistent lower affinity that compounds display towards the B-isoform with respect to the A-isoform. Also in this case, the molecules reported showed dissociation constants in the high-micromolar range for the B-isoform and this could be too high to completely saturate the enzyme *in vivo*.

We investigated the ability of the new set of compounds to interfere with bacterial growth. Minimal inhibitory concentration (MIC) in a medium with limiting cysteine, using the Gram-negative model organism, *E. coli* ATCC25922, was calculated for the most potent enzyme binders: compounds **5**, **12**, **16**, **24**, **33** and **47–50**. Unfortunately, none of the synthesised compounds was able to interfere significantly with bacterial growth (data not shown), which shows that the chemical modifications, although suitable to grant enzyme inhibition, did not allow to achieve sufficient inhibitor accumulation in bacteria.

## Conclusions

Small-molecule inhibitors of OASS enzymes have been developed since the inhibition of cysteine biosynthesis might be a valuable means to tackle down resistant infections. Despite the most potent molecule of the series exhibits a nanomolar affinity towards OASS isoforms, it was not able to interfere with *E. coli* growth in minimum medium, allegedly because of the poor permeability of the compound. Although the physicochemical properties that enable small molecule accumulation in the Gram-negative bacteria are yet not understood, previous analyses with different sets of compounds suggested that including nitrogen atoms in the structure is a fruitful option in order to obtain compounds able to cross Gram-negative cell wall. Therefore, we modified our hit molecule towards more polar derivatives by including in the structure nitrogen-containing groups or heterocycles. On the other side, the carboxylic acid group was replaced by several isosters. Most of the compounds synthesised were found to maintain good binding affinity in the biochemical assays, corroborating the rational design of the analogues and allowing additional body of SAR to be reported. Substitution of the carboxylic acid group by a sulphonamide not only was found to be tolerated, but a novel mechanism of partial inhibition, to be further investigated, was disclosed. The most potent enzyme binders, assayed on the Gram-negative model organism *E. coli* in minimum medium, did not show MIC lower than 256 μg/mL. It might be hypothesised that despite the increase of polarity through the introduction of nitrogen-containing functional groups is tolerated in terms of SAR, probably some features such as the negative charge of the carboxylic acid are detrimental for the accumulation of the compounds. Based on these novel SAR insights, further chemical modifications are currently ongoing in our laboratories.
